# Biomass pyrolysis liquid to citric acid via 2-step bioconversion

**DOI:** 10.1186/s12934-014-0182-4

**Published:** 2014-12-31

**Authors:** Zhiguang Yang, Zhihui Bai, Hongyan Sun, Zhisheng Yu, Xingxing Li, Yifei Guo, Hongxun Zhang

**Affiliations:** Department of Environmental and Municipal Engineering, Henan University of Urban Construction, Pingdingshan, 467036 China; Laboratory of Environmental Biotechnology, Research Center for Eco-Environmental Sciences, Chinese Academy of Sciences, Beijing, 100085 China

**Keywords:** Lignocellulosic biomass, Pyrolysis, Levoglucosan, Bio-conversion, Citric acid

## Abstract

**Background:**

The use of fossil carbon sources for fuels and petrochemicals has serious impacts on our environment and is unable to meet the demand in the future. A promising and sustainable alternative is to substitute fossil carbon sources with microbial cell factories converting lignocellulosic biomass into desirable value added products. However, such bioprocesses require tolerance to inhibitory compounds generated during pretreatment of biomass. In this study, the process of sequential two-step bio-conversion of biomass pyrolysis liquid containing levoglucosan (LG) to citric acid without chemical detoxification has been explored, which can greatly improve the utilization efficiency of lignocellulosic biomass.

**Results:**

The sequential two-step bio-conversion of corn stover pyrolysis liquid to citric acid has been established. The first step conversion by *Phanerochaete chrysosporium* (*P. chrysosporium*) is desirable to decrease the content of other compounds except levoglucosan as a pretreatment for the second conversion. The remaining levoglucosan in solution was further converted into citric acid by *Aspergillus niger* (*A. niger*) *CBX-209*. Thus the conversion of cellulose to citric acid is completed by both pyrolysis and bio-conversion technology. Under experimental conditions, levoglucosan yield is 12% based on the feedstock and the citric acid yield can reach 82.1% based on the levoglucosan content in the pyrolysis liquid (namely 82.1 g of citric acid per 100 g of levoglucosan).

**Conclusion:**

The study shows that *P. chrysosporium* and *A. niger* have the potential to be used as production platforms for value-added products from pyrolyzed lignocellulosic biomass. Selected *P. chrysosporium* is able to decrease the content of other compounds except levoglucosan and levoglucosan can be further converted into citric acid in the residual liquids by *A. niger*. Thus the conversion of cellulose to citric acid is completed by both pyrolysis and bio-conversion technology.

## Background

Concern with environmental issues such as global climate change has stimulated research into the development of more environmentally friendly technologies and energy sources [1-4]. Wood and agricultural residues like corn stover are abundant and easily accessible at relatively low costs. This sustainable biomass has considerable advantages over petroleum-based sources and can be a reliable future source of added value chemicals and energy. Fast pyrolysis of lignocellulosic biomass for liquids production is of particular concern, as it is one of the interesting ways to produce renewable fuel and fine chemical precursors [5-8]. Their thermochemical conversion into gases and liquids used in gas turbines or diesel engines is one way to utilize these renewable resources. Cellulosic biomass can also be efficiently and rapidly converted into a high yield of pyrolysate with levoglucosan in high concentration under appropriate pyrolysis conditions [9,10]. The levoglucosan has attracted many investigators because its production and potential use as a fermentative carbon and energy source in the fermentation industry would lead to possible commercial utilization of large quantities of cellulosic materials [11,12]. However, during the pyrolysis of cellulosic materials, various toxic compounds, including aromatic species, aldehydes, furan and furfuryl derivatives, are formed, which inhibit the growth and fermentation of microorganisms [12-14]. Thus an additional and complicated detoxification step using many chemical reagents is needed to remove the inhibitors and improve microorganism growth prior to fermentation. It is disadvantageous because the process has large reagent consumption and waste production.

*Phanerochaete chrysosporium* is a filamentous basidiomycete white rot fungus, which is the subject of many investigations due to its ability to mineralize lignin and other related molecules [15,16]. The mineralization process is due to its peroxidases including lignin peroxidases (LiP), manganese-dependent peroxidases (MnP) and laccases secreted during metabolism [17]. These peroxidases are powerful oxidants that can oxidize not only phenols and aromatic amines, but also a variety of other aromatic ethers and polycyclic aromatics with appropriate ionization potentials [18].

Only those microorganisms with a specific levoglucosan kinase can directly convert LG to valuable products [19]. Our previous studies show that *A. niger CBX-209* can grow well on purified levoglucosan under optimum temperature, pH, the concentration of levoglucosan and wheat bran in the medium [20,21]. In this study, the efficient utilization for pyrolysate is developed. The bio-conversion process can be carried out in two steps. Other compounds except LG are utilized and converted by *P. chrysosporium* in the first step, and then remaining LG can be directly converted to citric acid by *A. niger CBX-209* in the second step. Two-step direct bioconversion of LG is advantageous because it avoids chemical pretreatment. The pre-fermentation step will not require costly reagents, but it has another cost. For example, the construction of a holding tank for this step will increase the process cost.

## Results and discussion

### The components of corn stover

The properties of corn stover were described in Table 1 after drying at 100 ± 5°C. Corn stover predominantly contains cellulose (41.46%), hemicellulose (32.63%) and lignin (16.22%). As comparison the values of references were also listed in Table 1. The difference between them was mainly because of different sources.

**Table 1:** **Analysis data of corn stover**

**Components analysis (wt.%)**		**Elemental analysis (wt.%)**	
Cellulose	41.46 (37.5)^b^	C	48.80 (45.53)^c^
Hemicellulose	32.63 (20.8)^b^	H	6.15 (5.03)^c^
Lignin	16.22 (17.6)^b^	N	0.78 (0.78)^c^
Neutral detergent solutes	8.88 (−−)^b^	O^a^	44.27 (41.11)^c^
		S	-- (0.13)^c^
Ash	0.81 (−−)^b^		

^a^Calculated by difference, ^b^reference [22], ^c^reference [23], −− no data.

### The pyrolysis liquid

**Figure 1:**
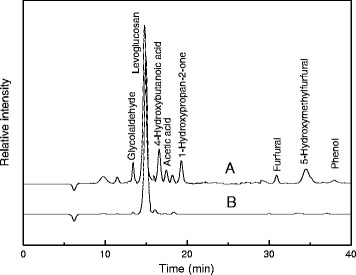
**HPLC of pyrolysis liquid. A**: crude pyrolysis liquid; **B**: liquid after conversion by *P. chrysosporium*.

Under this pyrolysis condition, the pyrolysis liquid yield is 68.8%, and its pH value is 2.8. The highest amount of separate organic compound groups analyzed in the water phase consisted of low molecular pyrolysis products, such as acetic acid, oxalaldehyde, levoglucosan, acetol, furan, furfuryl and other compounds (Figure 1). The main product was levoglucosan and its proportion of the total products was about 17.5%.

### First step bio-conversion

The previous results showed that both Pc and An can grow using pyrolysis oil as carbon source [24]. The pyrolysis liquid can be used as carbon source for *P. chrysosporium EBL0511* to grow at higher concentration, possibly because of higher concentration of hydrogen ion. The system of ligninolytic enzyme from *P. chrysosporium* is composed of lignin peroxidase, manganese peroxidase and glyoxal oxidase, which has a special degrading mechanism [25]. Similar to the oxidation of malonate by Manganese peroxidase (MnP) [26], for example, its biochemical reactions involved in the oxidation of glycolaldehyde by MnP are proposed as following 0ons (1, 2, 3, 4, 5, 6, 7, 8 and 9):

1 $$ \mathrm{H}\mathrm{O}\mathrm{C}{\mathrm{H}}_2\hbox{-} \mathrm{C}\mathrm{H}\mathrm{O} + \mathrm{M}\mathrm{n}\left(\mathrm{I}\mathrm{I}\mathrm{I}\right)+\frac{1}{2}{\mathrm{O}}_2\to \mathrm{H}\mathrm{O}\mathrm{C}{\mathrm{H}}_2^{\cdotp }+\mathrm{C}{\mathrm{O}}_2+{\mathrm{H}}^{+}+\mathrm{M}\mathrm{n}\left(\mathrm{I}\mathrm{I}\right) $$

2 $$ \mathrm{H}\mathrm{O}\mathrm{C}{\mathrm{H}}_2^{\cdotp }+{\mathrm{O}}_2\to \mathrm{H}\mathrm{O}\mathrm{C}{\mathrm{H}}_2\mathrm{O}{\mathrm{O}}^{\cdotp } $$

3 $$ \mathrm{H}\mathrm{O}\mathrm{C}{\mathrm{H}}_2\mathrm{O}{\mathrm{O}}^{\cdotp }+\mathrm{M}\mathrm{n}\left(\mathrm{I}\mathrm{I}\right)+{\mathrm{H}}^{+}\to \mathrm{H}\mathrm{O}\mathrm{C}{\mathrm{H}}_2\mathrm{O}\mathrm{O}\mathrm{H}+\mathrm{M}\mathrm{n}\left(\mathrm{I}\mathrm{I}\mathrm{I}\right) $$

4 $$ \mathrm{H}\mathrm{O}\mathrm{C}{\mathrm{H}}_2\mathrm{O}\mathrm{O}\mathrm{H}+2\mathrm{M}\mathrm{n}\left(\mathrm{I}\mathrm{I}\right)\;\overset{\mathrm{MnP}}{\to}\mathrm{H}\mathrm{O}\mathrm{C}\mathrm{H}\mathrm{O}+{\mathrm{H}}_2\mathrm{O}+2\mathrm{M}\mathrm{n}\left(\mathrm{I}\mathrm{I}\mathrm{I}\right) $$

5 $$ \mathrm{H}\mathrm{O}\mathrm{C}{\mathrm{H}}_2\mathrm{O}{\mathrm{O}}^{\cdotp }+{\mathrm{O}}_2\to {\mathrm{H}}_2\mathrm{O}+\mathrm{C}{\mathrm{O}}_2+{\mathrm{O}}_2^{\cdotp \hbox{-} }+{\mathrm{H}}^{+} $$

6 $$ \mathrm{HOCHO}+\mathrm{M}\mathrm{n}\left(\mathrm{I}\mathrm{I}\right)+\frac{1}{2}{\mathrm{O}}_2\to {\mathrm{H}}_2\mathrm{O}+\mathrm{C}{\mathrm{O}}_2^{\cdotp -}+\mathrm{M}\mathrm{n}\left(\mathrm{I}\mathrm{I}\mathrm{I}\right) $$

7 $$ \mathrm{C}{\mathrm{O}}_2^{\cdotp -}+{\mathrm{O}}_2\to \mathrm{C}{\mathrm{O}}_2+{\mathrm{O}}_2^{\cdotp -} $$

8 $$ {\mathrm{O}}_2^{\cdotp -}+2{\mathrm{H}}^{+}+\mathrm{M}\mathrm{n}\left(\mathrm{I}\mathrm{I}\right)\to {\mathrm{H}}_2{\mathrm{O}}_2+\mathrm{M}\mathrm{n}\left(\mathrm{I}\mathrm{I}\mathrm{I}\right) $$

9 $$ {\mathrm{H}}_2{\mathrm{O}}_2+\mathrm{M}\mathrm{n}\left(\mathrm{I}\mathrm{I}\right)\overset{\mathrm{MnP}}{\to }{\mathrm{H}}_2\mathrm{O}+2\mathrm{M}\mathrm{n}\left(\mathrm{I}\mathrm{I}\mathrm{I}\right) $$

Figure 1 shows the changes in components after 7-days liquid cultures. *P. chrysosporium EBL0511* is effective for degradation of pyrolysis liquid. Other components except levoglucosan were almost completely degraded by it.

### Second step bio-conversion

**Figure 2:**
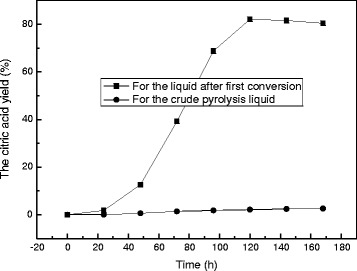
**Course of the fermentation for the liquid after first conversion and the crude pyrolysis liquid.**

The previous results showed that some components of pyrolysis liquid inhibited the growth of *A. niger*, but levoglucosan can be used as carbon source for it. Reports [27,28] have found some microorganisms can assimilate levoglucosan, one of the components of pyrolysis liquid by phosphorylation to glucose 6-phosphate in the presence of magnesium ion and ATP (10):

The liquid after the first conversion was further fermented to citric acid by *A. niger CBX-209*. The time course of citric acid fermentation is shown in Figure 2. Very little citric acid is produced for crude pyrolysis liquid. It means that some components of pyrolysis liquid have toxic effects on the growth of *A. niger CBX-209*. However the citric acid yield reaches 82.1% when using pyrolysis liquid after the first conversion. Accumulation of citric acid by *A. niger CBX-209* could be divided into three steps. During the first step from inoculation to 24 h, very little citric acid was produced due to lower biomass. In the second phase that lasted from 24 to 72 h, the citric acid yield increased from about 1.8% to 82.1% rapidly with increasing biomass. The highest citric acid yield is slightly lower than the yield (87.5%) when using purified levoglucosan as the sole carbon source [21]. In the third step, after 72 h, the yield was maintained at almost the same level. The high yield of citric acid by *A. niger CBX-209* should be attributed to low molecular weight of LG and highly active microbe.

## Conclusions

To obtain citric acid from corn stover pyrolysis liquid, serial 2-step conversion is established. In the first step conversion by *P. chrysosporium*, other compounds except levoglucosan are metabolized. Thus it can be used as a pretreatment for the second conversion. In the second step the liquids containing levoglucosan are further converted into citric acid by *A. niger CBX-209*. The result of serial conversion of corn stover pyrolysis liquid shows that the citric acid yield can reach 82.1% based on the levoglucosan in the pyrolysis liquid.

## Materials and methods

### Materials

The corn stover used in all the experiments was obtained from Juxian field (Shandong province, China). It was oven dried at 100 ± 5°C for 12 h. The original length was between 2 and 50 mm. It was partly ball-milled and screened to achieve a size of less than 20 mesh and larger than 48 mesh prior to the pyrolysis. The sample was analyzed by the elemental analyzer to determine the major elements such as carbon (C), hydrogen (H) and nitrogen (N). The properties were described after drying at 100 ± 5°C. The determination of components was performed by the following procedure: a dried sample (1 g) – neutral detergent residue – acid detergent residue – 72 wt.% sulfuric acid detergent residue – ashing, where neutral detergent is 100 ml water solution containing 1.86 g EDTA, 0.68 g Na_2_B_4_O_7_·10H_2_O, 3 g sodium dodecyl sulfate, 1 ml glycol ether and 0.456 g Na_2_HPO_4_, acid detergent is 100 ml 2 mol/L HCl solution containing 2 g Cetyl trimethyl ammonium bromide. Lignocellulose contents of all samples were determined by boiling in 100 ml detergent solution for 1 hr under reflux. Then these residues were filtered, washed with hot water and acetone, dried at 100°C for 8 hr and weighed.

### Pyrolysis apparatus and procedures

The self-designed pyrolyzer has been introduced and used in our previous works [29,30]. For the present work, briefly, the reaction system is evacuated by a rotary pump with an ultimate pressure of 6 × 10^−2^ Pa and a pumping speed of 15 L/s. Reactor is loaded with a batch of feedstock (50 g), sealed and evacuated, then inserted into the furnace. Samples are pyrolyzed at a pre-set oven temperature 390°C. Condensable product is collected in an ice-water condenser situated between the pyrolysis oven and the filter. At desired reaction time (here is 40 min), reactor is pulled out of the furnace and water-cooled and then after release of vacuum the residual is reweighed. The feedstock for pyrolysis is corn stover.

The pyrolysis liquid and its bio-conversion products are quantitatively diluted with distilled water and analyzed by a HPLC system (GRE-3A Shimadzu) equipped with a Waters Model 401 refractive index detector and a Transgenomic ICSep ICEORH- 801 column (300 mm × 6.5 mm), the injection volume is 10 μl and the column temperature is maintained at 48°C. The eluent is 0.005 mol/L sulfuric acid with flow rate of 0.6 ml/min. Products are assayed by comparing the peak area for the sirup with that of the standard samples from Sigma. HPLC peaks are labeled based on the identical retention time with the standard samples. Each experiment is repeated three times and the mean of them is used for the analysis.

### Microorganisms and medium

Microorganisms: *P. chrysosporium EBL0511* (Pc) and *A. niger CBX-209* (An) are used. Both of them are from our lab. Initially, *P. chrysosporium EBL0511* was obtained from China General Microbiological Culture Collection Center. The mutant, *A. niger CBX-209*, was derived by γ-ray irradiation of spores of parent strain *CBX-2*, which has high citric acid productivity using starch as carbon and energy source at an industrial scale [20,21]. The solid medium was prepared by Potato medium (PM). PM contained 20% Potato extract, 0.2% Glucose and 1.5% Agar. Blank medium (BM) used only agar and pyrolysis oil. The potato extract (Wako) was purchased from Express Technology Co., Ltd.

*P. chrysosporium* was used in the first step. The mutant, *A. niger CBX-209* with high citric acid productivity was used in the second step. The seed culture had a composition of 20 g/L glucose, 10 g/L yeast extract, 1 g/L K_2_HPO_4_, 1 g/L MgSO_4_ · 7H_2_O and 20 g/L peptone. The supernatant was collected by centrifugation from the liquid after first conversion, added 3% wheat bran, and go into the autoclave then inoculate with *A. niger CBX-209*. The fermentation media incubated at 35°C on a rotary shaker at 400 rpm for 5 days, and used for the analyses. Three replicates were performed for each fermentation experiment and each yield was the mean of three replicates.

The pyrolysis liquid needs to be diluted with equal amount of distilled water and then neutralized using Na_2_CO_3_ to pH 6.0 before it was added into mediums.

## Abbreviations

LG: Levoglucosan
MnP: Manganese peroxidase
Pc: *Phanerochaete chrysosporium*
An: *Aspergillus niger*
PM: Potato medium
BM: Blank medium
